# The Influence of Age on the Development of Dental Caries in Children. A Radiographic Study

**DOI:** 10.3390/jcm10081702

**Published:** 2021-04-15

**Authors:** Abel Emanuel Moca, Luminița Ligia Vaida, Bianca Maria Negruțiu, Rahela Tabita Moca, Bianca Ioana Todor

**Affiliations:** 1 Department of Dentistry, Faculty of Medicine and Pharmacy, University of Oradea, 1 Universității Street, 410087 Oradea, Romania; abelmoca@yahoo.com (A.E.M.); biancaioana.todor@gmail.com (B.I.T.); 2 Clinical Emergency County Hospital Oradea, 37 Republicii Street, 410167 Oradea, Romania; rahelamoca@gmail.com

**Keywords:** chronological age, dental age, first permanent molars, dental caries

## Abstract

Dental caries is a chronic disease that can be influenced by a multitude of factors. Poor oral hygiene and unhealthy eating habits are the most incriminating factors in the onset of dental caries, but age has been proven to impact the disease. The aim of this study was to find correlations between age and the evolution of dental caries in the first permanent molars in children. The retrospective study was conducted based on 400 panoramic radiographs, belonging to Romanian children between the ages of 6 and 14 years. All first permanent molars were investigated, and the carious lesions were classified according to their depth. The chronological age was calculated by subtracting the patient’s date of birth from the date when the radiograph was performed, while for the assessment of dental age, the Demirjian method was used. The gender of the patients did not significantly influence the number of superficial, medium, and deep carious lesions. Most of the identified carious lesions were superficial. Regarding correlations between age and dental caries, there was an association between the decrease in the chronological age and the increase in the number of superficial carious lesions on the first permanent molars and also an association between the increase in the chronological age or dental age and the increase in the number of medium and deep carious lesions on the first permanent molars. Age can impact the development of dental caries in first permanent molars.

## 1. Introduction

Dental caries is a multifactorial disease [1] and the most commonly diagnosed chronic disease in children [2]. It remains a major public health problem in most industrialized countries [3]. Despite its high global prevalence and negative impact on people [2], it is a widely neglected public health issue [4].

Poor oral hygiene [5] and unhealthy eating habits [6] are the most common incriminating factors in the onset and development of tooth decay, but factors such as low socioeconomic status, low level of education, or even orthodontic treatment, have been shown to be important in predicting the prevalence of caries [7,8]. The detection of risk factors must be done from an early age due to the immediate and long-term harmful effects that dental caries can produce [9]. Once installed, the evolution is fast, with appropriate treatment ranging from minimally invasive methods [10,11] to treatments performed under general anesthesia [9,12].

Dental caries can affect both primary and permanent dentition. Furthermore, if a child experiences tooth decay in the primary dentition, the probability of experiencing the same pathology in the permanent dentition grows [13]. The first permanent teeth to erupt in the oral cavity are, traditionally, the first permanent molars, which often erupt before the age of 6 years [14]. Their complex occlusal morphology, the eruption at young ages, as well as the other incriminating factors associated with the development and the evolution of dental caries, makes them prone to the early onset of the carious disease [15].

The influence of age on the occurrence of dental caries involves various changes that arise at different periods of time, but it can also be a direct determinant of the disease [16]. Age determination can be based on the assessment of chronological age, dental age, or skeletal age [17]. Skeletal age can be investigated using various methods involving the visualization of different bone structures, such as the clavicle, the iliac bone, the femoral head, the bones of the hand and wrist, or the cervical vertebrae [18]. Dental age is easy to determine using the patient’s panoramic radiograph. Although many radiological methods for the assessment of the dental age have been developed, Demirjian’s method is the most widely used [19].

The aim of this study was to establish if chronological age and dental age have any influence on the occurrence of dental caries in first permanent molars in a sample of Romanian children.

## 2. Materials and Methods

### 2.1. Sample Selection

This retrospective radiographic study was performed on digital panoramic radiographs belonging to children from North-Western Romania and was conducted between the 20th of October 2020 and the 1st of March 2021. The panoramic radiographs were collected from three private dental offices in Oradea, Romania, and were taken using the Soredex Cranex Novus Panorex system. All radiographs were considered a necessary investigation for the completion of the required dental treatments and were not requested only for this research.

We included panoramic radiographs belonging to patients aged between 6 and 14 years, patients who needed a panoramic radiograph for the diagnosis and treatment of dental conditions, patients for whom the date of birth was known, and patients who had all four permanent first molars erupted.

The panoramic radiographs excluded from the study belonged to uncooperative patients whose behavior did not allow the completion of a proper panoramic radiograph resulting in an unclear radiographic image, patients from other countries, patients with local or general pathologies that could influence the eruption of permanent teeth, and patients who had at least one missing erupted or unerupted permanent tooth in the lower left dental arch. 

Initially, 530 panoramic radiographs were selected, but after applying the exclusion criteria, 400 panoramic radiographs were kept in the study. A total of 1600 first permanent molars were investigated (four first permanent molars for each patient).

### 2.2. Investigation of Carious Lesions

**Figure 1:**
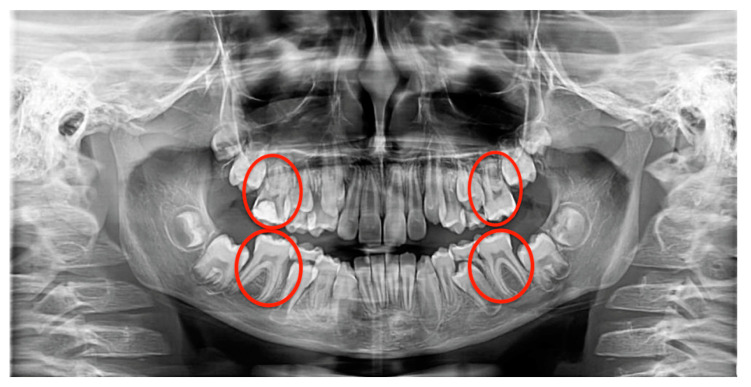
Panoramic radiograph with the first permanent molars highlighted.

**Figure 2:**
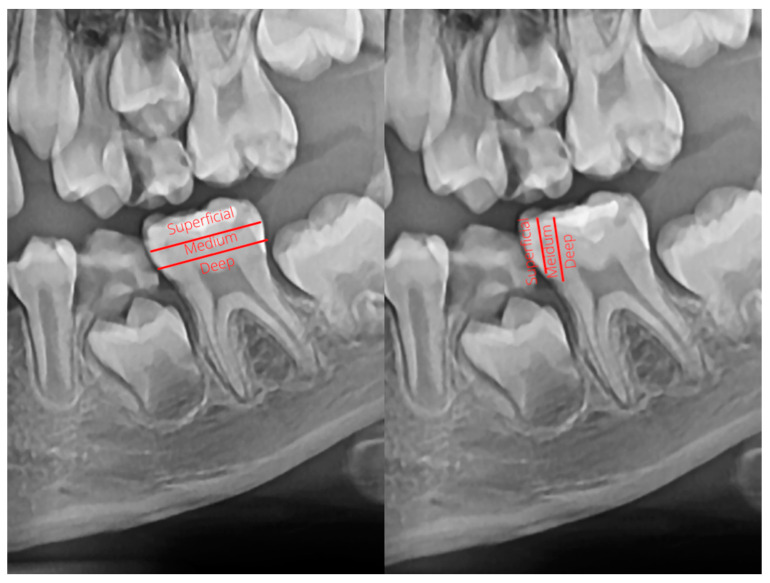
Classification of dental caries according to their depth.

All erupted first permanent molars were radiologically investigated (Figure 1). The coronal part of the first permanent molars, extending from the pulp chamber to the enamel layer, was horizontally and vertically divided into three thirds. The treated and untreated dental caries were classified according to their depth into superficial carious lesions (extended to the outer third of the dentin), medium carious lesions (extended to the inner third of the dentin), and deep carious lesions (extended near the pulp chamber or with involvement of the pulp chamber). As such, if a caries or restoration extended under the enamel or in the outer third of the dentin, with a minimal dentin involvement, it was considered superficial. If a caries or restoration extended to the inner third of the dentin, with a highly visible and thick intact dentin layer between the caries and the pulp chamber, it was considered medium. Caries or restorations extended close to the pulp chamber, with a thin dentin layer between the caries and the pulp chamber, or those concerning the pulp chamber, were classified as deep (Figure 2). Only the mesial, distal, and occlusal surfaces of the molars were analyzed, as the vestibular and lingual surfaces are difficult to examine on panoramic radiographs.

### 2.3. Chronological and Dental Age Assessment

**Figure 3:**
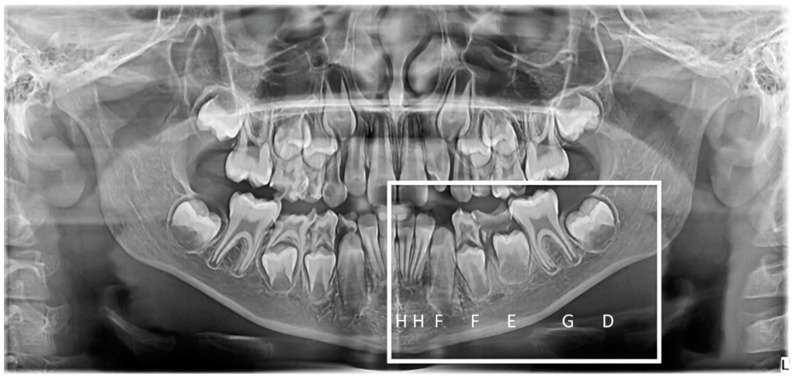
Exemplification of Demirjian’s method for dental age assessment.

The chronological age was calculated by subtracting the patient’s date of birth from the date when the radiograph was performed. For the assessment of the dental age, the Demirjian method was used (Figure 3). This analyses the development stages of all seven permanent teeth of the lower-left dental arch and assigns each tooth a score. After all the obtained scores are added, the final result is transformed into dental age, based on the tables envisioned by Demirjian et al. (1974) [20]. The examinations were performed by a single investigator to avoid inter-operator bias (A.E.M.).

### 2.4. Statistical Analysis

Statistical analysis was performed by using IBM SPSS software, version 20 (IBM, Chicago, IL, USA). Quantitative variables were tested for distribution using the Shapiro–Wilk test and were expressed as mean values with standard deviations (or medians with interpercentile intervals, depending on the distribution) and the categorical variables were expressed in absolute or percentage form. The independent quantitative variables were tested using the Mann–Whitney U test, as their distribution was non-parametric, and the existing correlations were proved using Spearman’s Rho correlation coefficient.

### 2.5. Ethical Considerations

The study was conducted in accordance with the 1964 Declaration of Helsinki and its later amendments and was approved by the Research Ethics Committee of the University of Oradea (No.7/15.10.2020).

## 3. Results

Regarding the distribution of the patients related to gender, 231 (57.8%) radiographs belonged to female patients, and 169 (42.2%) panoramic radiographs belonged to male patients. As such, a total of 1600 first permanent molars were radiologically investigated, of which 924 belonged to female patients and 676 belonged to male patients. The mean chronological age was 9.9 ± 1.8 years, with a median of 9.8 years and a range of 6.5 and 13.9 years, and the mean value of dental age was 11.3 ± 2.2 years, with a median of 11.2 years and a range between 7.2 and 16 years.

**Figure 4:**
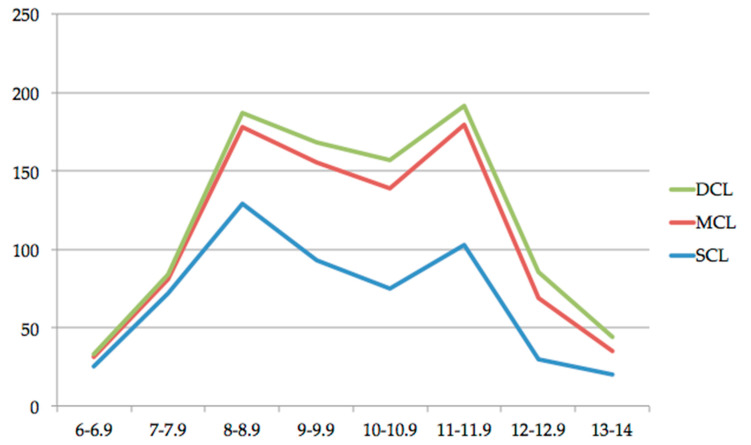
Distribution of superficial carious lesions (SCL), medium carious lesions (MCL), and deep carious lesions (DCL) according to the chronological age of the patients (expressed in years).

**Figure 5:**
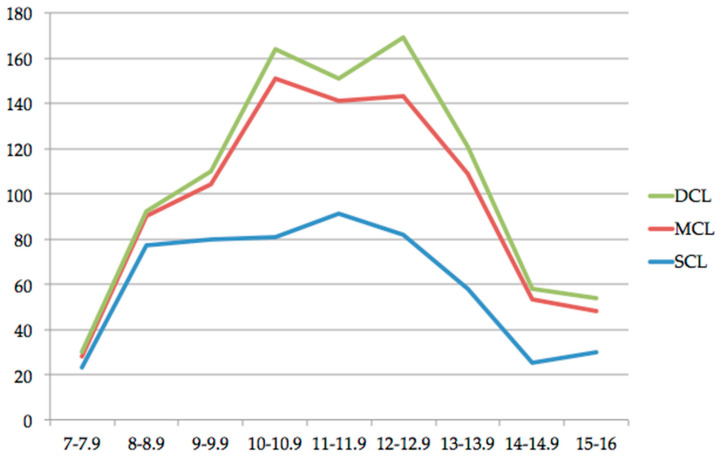
Distribution of superficial carious lesions (SCL), medium carious lesions (MCL), and deep carious lesions (DCL) according to the dental age of the patients (expressed in years).

Treated and untreated carious lesions of the first permanent molars were investigated and counted. Most of the identified carious lesions were superficial (*n* = 547, 57.6%), followed by medium carious lesions (*n* = 320, 33.7%) and deep carious lesions (*n* = 82, 8.7%). A detailed distribution is given in Table 1, Figure 4, and Figure 5. 

The comparison between the number of superficial, medium, and deep carious lesions related to the gender of the patients showed that the distribution of the number of carious lesions was non-parametric in all three types of carious lesions for both genders. According to the Mann–Whitney U test, the differences were not statistically significant, so the gender of the patients did not significantly influence the number of superficial, medium, and deep carious lesions (Table 2).

The data in Table 3 represent the correlation between chronological age and the number of superficial, medium, and deep carious lesions. Both variables had a non-parametric distribution according to the Shapiro–Wilk test. In the case of superficial carious lesions, the observed correlation was significant, negative and of a very low degree, which shows that there was an association between the decrease in chronological age and the increase in the number of superficial carious lesions on the first permanent molars. For medium and deep carious lesions, the observed correlation was significant, positive and of a very low degree, which shows that there was an association between the increase in chronological age and the increase in the number of medium and deep carious lesions on the first permanent molars.

The data in Table 4 represent the correlation between dental age and the number of superficial, medium, and deep carious lesions. Both variables had a non-parametric distribution according to the Shapiro–Wilk test. For superficial carious lesions, the observed correlation was insignificant, so dental age did not significantly influence the number of superficial carious lesions on the first permanent molars. However, for medium and deep carious lesions, the observed correlation was significant and positive of a very low degree, which shows that there was an association between the increase in dental age and the increase in the number of medium and deep carious lesions on the first permanent molars.

## 4. Discussion

Panoramic radiographs are complementary methods of examination widely used in dentistry [21], which are useful in combination with oral examinations to identify oral health problems [22]. The use of panoramic radiography was preferred for this research because it allows both the assessment of the patient’s dental age by the Demirjian method [20,23] and the visualization of the patient’s entire dentition. Therefore, avoiding any additional radiation exposure, the radiation dose required for a digital panoramic radiograph is low [24]. An important advantage of using radiological examination, to the detriment of oral examination, is dependent on the current epidemiological context. The COVID-19 pandemic has led to a decline in dental services provided for pediatric patients [25] with oral examinations performed on large samples being difficult due to strict infection control measures [26]. At the same time, periapical radiographs and bite-wing radiographs were avoided in line with the policies and recommendations issued by the competent authorities in Romania.

Our sample selection included patients between the ages of 6 and 14 years. The inferior limit of 6 years was selected because the first permanent molar is expected to erupt or be completely erupted by the age of 6, while the second permanent molar is expected to be completely erupted by the age of 14 years [27].

Regarding the assessment of the dental age, the Demirjian method was used because even though it may indicate differences between the chronological age and dental age, it remains the most used method for determining the dental age [28]. A number of studies suggest that the method tends to overestimate the age in different populations and that the values need to be adapted for each investigated group [29,30]. In the studied sample of patients, differences were found between dental age and chronological age, but no statistical tests were performed, since the purpose of the study was not to analyze the accuracy of the Demirjian method.

First permanent molars were selected for radiographic inspection because, usually, they are the earliest permanent teeth to erupt in the oral cavity, at around the age of 6 [31], with variations related to topography and gender [32]. At the same time, the occlusal morphology of the first permanent molars makes them susceptible to dental caries [33] immediately after eruption [34]. The superficial, medium, and deep, treated and untreated carious lesions in all four first permanent molars were also identified in this study with most of the carious lesions identified being superficial. Although the DMFT index is preferred for the assessment of carious activity [35], the method we used was suitable for panoramic radiographs.

The main novelty of the present study was the investigation of possible correlations between age (chronological and dental) and carious experience of the first permanent molars. We identified only one article that correlates the chronological age to the carious activity of the first permanent molars, but we did not identify any article that correlates the dental age to the carious activity of the first permanent molars. In our study, the correlations were not made separately for each gender because no significant differences were identified between the number of carious lesions (superficial, medium, and deep) between boys and girls, even if other authors indicated a higher prevalence of caries in female patients [36]. Correlations were identified between the increase in chronological and dental age and the increase in the number of medium and deep caries, as well as between the decrease in chronological age and the increase in the number of superficial caries. This suggests that carious onset occurs at an early age and continues to aggravate over time. The results are similar to those obtained by Al-Samadani et al. (2012), who conducted research on a sample of 432 school children, with ages between 9 and 12 years. The authors discovered a high carious activity in the first permanent molars and a correlation between the increase in carious prevalence and the increase in age [15]. Bernabé and Sheiham (2014) concluded that caries levels increase with age and remain a major problem in the adult population [37]. Moreover, a high carious level in the primary dentition raises the probability of a high carious level in the permanent dentition [38].

This study, however, has its limitations. The number of panoramic radiographs investigated was 400. Larger samples are necessary for more accurate results. Caries detection was based on the investigation of panoramic radiographs, and no clinical examinations were performed. Ideally, a good oral examination is of much aid in the early detection of dental caries. At the same time, only the first permanent molars were examined, but other permanent teeth could be examined too, so that a more detailed conclusion could be drawn.

## 5. Conclusions

The increase in chronological and dental age can be considered an indicator of a more intense carious experience of the first permanent molars and for medium and deep carious lesions. For lower chronological ages, superficial carious lesions are more commonly detected.

## Figures and Tables

**Table 1:** Distribution of identified carious lesions.

Type of Treated or Untreated Carious Lesion	Investigated Permanent Molar (*n*, %)	Treated or Untreated Carious Lesions (*n* = 949)
Superficial carious lesions	First permanent upper-right molar	125 (13.2%)
	First permanent upper-left molar	120 (12.6%)
	First permanent lower-right molar	148 (15.6%)
	First permanent lower-left molar	154 (16.2%)
Medium carious lesions	First permanent upper-right molar	76 (8%)
	First permanent upper-left molar	60 (6.3%)
	First permanent lower-right molar	97 (10.2%)
	First permanent lower-left molar	87 (9.2%)
Deep carious lesions	First permanent upper-right molar	18 (1.9%)
	First permanent upper-left molar	17 (1.8%)
	First permanent lower-right molar	20 (2.1%)
	First permanent lower-left molar	27 (2.9%)

**Table 2:** Comparison between the number of superficial, medium, and deep carious lesions related to gender.

Type of Treated or Untreated Carious Lesion	Gender	Mean Number ± SD	Median (IQR)	Medium Rank	*p* *
Superficial carious lesions	Female (*p* < 0.001 **)	1.35 ± 1.524	1 (0–2)	195.33	0.273
	Male (*p* < 0.001 **)	1.49 ± 1.476	1 (0–2.5)	207.57	
Medium carious lesions	Female (*p* < 0.001 **)	0.8 ± 1.17	0 (0–1)	200.78	0.949
	Male (*p* < 0.001 **)	0.87 ± 1.303	0 (0–2)	200.12	
Deep carious lesions	Female (*p* < 0.001 **)	0.26 ± 0.799	0 (0–0)	201.34	0.770
	Male (*p* < 0.001 **)	0.18 ± 0.542	0 (0–0)	199.36	

* Mann–Whitney U test; ** Shapiro–Wilk test; SD—Standard Deviation; IQR—Interquartile range.

**Table 3:** Correlations between chronological age and superficial, medium, and deep carious lesions.

Type of Treated or Untreated Carious Lesion	Correlation	*p* *
Superficial carious lesions	CA (*p* < 0.001 **) × SCL (*p* < 0.001 **)	0.011, R = −0.127
Medium carious lesions	CA (*p* < 0.001 **) × MCL (*p* < 0.001 **)	<0.001, R = 0.220
Deep carious lesions	CA (*p* < 0.001 **) × DCL (*p* < 0.001 **)	0.002, R = 0.153

* Spearman´s rho Correlation Coefficient; ** Shapiro-Wilk Test; CA—Chronological Age; SCL—Superficial Carious Lesions; MCL—Medium Carious Lesions; DCL—Deep Carious Lesions.

**Table 4:** Correlations between dental age and superficial, medium, and deep carious lesions.

Type of Treated or Untreated Carious Lesion	Correlation	*p* *
Superficial carious lesions	DA (*p* < 0.001 **) × SCL (*p* < 0.001 **)	0.076, R = −0.089
Medium carious lesions	DA (*p* < 0.001 **) × MCL (*p* < 0.001 **)	<0.001, R = 0.181
Deep carious lesions	DA (*p* < 0.001 **) × DCL (*p* < 0.001 **)	0.003, R = 0.146

* Spearman´s rho Correlation Coefficient; ** Shapiro–Wilk test; DA—Dental Age; SCL—Superficial Carious Lesions; MCL—Medium Carious Lesions; DCL—Deep Carious Lesions.

## Data Availability

The data presented in this study are available on request from the corresponding author. The data are not publicly available due to privacy reasons.
